# What is favourable conservation status?: A systematic map protocol

**DOI:** 10.1186/s13750-025-00356-7

**Published:** 2025-02-17

**Authors:** Alice M. Oswald, Natasha Mannion, Stephen G. Willis, Philip A. Stephens, Philip J.K. McGowan

**Affiliations:** 1 Department of Biosciences, University of Durham, Durham, England; 2 School of Natural and Environmental Sciences, Newcastle University, Newcastle upon Tyne, England

**Keywords:** Biodiversity conservation, Conservation policy, Favourable reference value, Species conservation, Resilient populations

## Abstract

**Background:**

Favourable Conservation status (FCS) is the overarching goal of the Habitats and Birds Directives, in which it is described as the situation in which a habitat or species is thriving throughout its natural range and is expected to continue to thrive. However, despite being introduced over thirty years ago, FCS has not been widely adopted as a conservation assessment framework. This systematic map aims to collate and characterise evidence to understand 1) how the term FCS is used in the literature, and 2) the context of its applications in policy and practice. Specifically, we ask the question: how is FCS defined and how has it been applied in policy and practice? This review will contribute to the field by providing the first systematic evidence synthesis on FCS, both as a concept and as a practical application, and will review the broader applicability of FCS beyond Member State reporting obligations.

**Methods:**

A systematic search of the literature will be conducted to collate and categorise evidence on the definitions and applications of FCS and barriers to its implementation. The literature will be screened in two stages to assess relevance, firstly by titles and abstracts and secondly by the full-texts. Studies will be assessed against eligibility criteria pertaining to the components of the question. Coded data will be extracted from the relevant studies and used in a narrative synthesis to summarise the evidence in a discussion, complemented by descriptive statistics and visual aids.

**Supplementary Information:**

The online version contains supplementary material available at 10.1186/s13750-025-00356-7.

## Background

Quantifiable and robust conservation targets are necessary to safeguard biodiversity effectively amidst the unprecedented biodiversity loss observed in the last half-century [1]. While traditional conservation approaches have played a critical role in protecting biodiversity, there is a growing recognition of the need for even more ambitious and proactive strategies that not only prevent extinction but also ensure that species and habitats thrive in the long-term [2, 3]. One example of such an approach is the concept of Favourable Conservation Status (FCS).

The foundations of FCS were established in the Bonn Convention 1979 and later adopted into EU policy as an overarching goal of the Habitats Directive 1992 [4]. Although the term ‘FCS’ is not explicitly used in the Birds Directive 1979, its goals are aligned with the concept of FCS [5] and are equated to the achievement of FCS for birds in the EU [6, 7]. The Habitats and Birds Directives are referred to collectively as the Nature Directives.

The Habitats Directive states that the conservation status of a habitat will be considered as favourable when:

- its **natural range** and areas it covers within that range are stable or increasing, and.
- the specific **structure and functions** which are necessary for its long-term maintenance exist and are likely to continue to exist for the foreseeable future, and.
- the conservation status of its **typical species** is favourable (as defined below).

and the conservation status of a species will be considered as favourable when:

- **Population dynamics** on the species indicate that it is maintaining itself on a long-term basis as a viable component of its natural habitats:
- The **natural range** of the species is neither being reduced nor is likely to be reduced for the foreseeable future:
- There is, and will probably continue to be, a **sufficiently large habitat** to maintain its populations on a long-term basis.

The quantitative measures derived from the Habitats Directive FCS definition are known as Favourable Reference Values (FRVs). FRVs define a minimum threshold for a species or habitat to be in an FCS [8]. FRVs include the Favourable Reference Range (FRR), Favourable Reference Population (FRP) and the Favourable Reference Area (FRA). Other independent parameters are considered with FRVs to produce an overall FCS definition for a particular species or habitat, including the habitat for the species and the structure and function of habitats (Table 1).

A 2018 European Commission report described the diverse approaches taken by EU Member States in setting FRVs and assessing FCS [7]. However, key findings from previous reviews indicate that a lack of a standardised methodology has resulted in varied interpretations and inconsistent assessments of FCS [9, 10]. This barrier highlights one of several challenges in translating the FCS concept from policy into practice [11–13] and the need for a comprehensive understanding of the existing evidence on the FCS landscape.

**Table 1:** Favourable reference values (*) and other parameters used to develop an overall favourable conservation status assessment for species and habitats. Compiled from [7]

Species	Habitat
Favourable Reference Range*	Favourable Reference Range*
Favourable Reference Population*	Favourable Reference Area*
Habitat for the species	Structure and function

### Policy and practice in the context of FCS

It is important to clarify what is meant by conservation policy and practice in the current context. Conservation policy provides a framework of plans and guidelines for biodiversity conservation, which outline actions and considerations for implementation [14]. Policies often come with some degree of national or international legal obligation for monitoring and reporting. Evidence from conservation science is one of many sources of information that informs these policies [15], which can then influence practice through specific conservation actions [16]. The conservation policy of interest in this review is the requirement for the maintenance of FCS for species and habitats outlined in the annexes of the Nature Directives. An example of conservation action in this context is the development of an FCS definition for a particular species or habitat that informs on the ground management of a site or species to achieve or maintain FCS. For example, defining FCS for a species such as the Eurasian beaver (*Castor fiber*) in England includes determining required population numbers and the extent and quality of habitat, which then informs targeted conservation strategies and actions such as reintroduction programmes and habitat restoration [17].

### Current and potential applications of FCS in policy and practice

The current European policy framework requires Member States to report to the Nature Directives every six years as stipulated by Article 17 of the Habitats Directive and Article 12 of the Birds Directive [18]. Although the UK is no longer an EU Member State, FCS remains a relevant piece of legislation for the UK as it is now reflected in EU Exit Habitats Regulations (*UK Government* 2019), which requires reporting under Regulation 9A [19]. Indeed, some notable applications of FCS can be seen in the work of Natural England, a UK public body that advises the government Department of Environment, Food and Rural Affairs (DEFRA). Natural England has published 39 FCS definitions for species and habitats to date [20]. Licensing decisions made by Natural England, such as those related to mitigation licenses for developments, are subject to an ‘FCS test’ to assess whether they will affect the conservation status of the species or habitat [21]. For example in England, FCS is the primary evaluation criterion in district-level licensing for Great Crested Newts (*Triturus cristatus*) [22]. FCS is also used in decisions relating to Habitat regulation assessments [23] and is the management objective of all Special Areas of Conservation (SACs) [24]. These implementations demonstrate how FCS can be embedded into existing policy frameworks to directly influence action and highlight the potential for widening applications in other bodies, countries and policy frameworks. FCS has significant potential to influence biodiversity conservation by contributing to biodiversity indicators within the goals of the Kunming-Montreal Global Biodiversity Framework [25]. The aim to increase the abundance of native wild species to ‘healthy and resilient’ levels within Goal A and Target 4 (Halt Species Extinction, Protect Genetic Diversity, and Manage Human-Wildlife Conflicts) depends on clearly defining those terms akin to the ‘favourable’ population sizes described in the Habitats Directive. The new EU Nature Restoration law signed in August 2024 will play a crucial role in aligning EU efforts with the Nature Directives and the global Kunming-Montreal GBF commitments [26].

Here we have introduced some of the potential areas where FCS can be applied to support meaningful conservation actions by a multitude of decision-makers and practitioners at different political and geographic scales. FCS has value as a conservation assessment tool applicable to any species or habitat of conservation interest beyond EU policy or reporting obligations. It provides an ambitious and quantifiable vision of what the ‘favourable’ state is, serving as a useful measure of not just how close a species or habitat is to extinction but also assessing if existing conservation strategies are delivering enough to achieve FCS for that species or habitat. However, FCS has not yet been utilised to the full extent of its potential.

Several studies have investigated the implementation, legitimacy and effectiveness of the Nature Directives to protect biodiversity in Europe [27–29] and whilst FCS is well-defined in some countries [8, 30], fewer studies have assessed the utility of FCS as a concept [31] and fewer again have demonstrated how the FCS concept is translated into conservation action [12], resulting in applications of FCS being described as rare [32].

Therefore, the question still largely remains: what is FCS, and why has its implementation across the EU remained limited, despite its introduction into EU policy over three decades ago? Additionally, it is essential to map out the evidence on the different scales at which FCS is being studied because FCS is deeply scale-dependent, and definitions may vary at different geographic scales [33]. To answer these questions, an exploration into the current and potential applications of FCS in policy and practice is required. However, a systematic evidence synthesis has not been conducted to gather evidence that demonstrates a gap between the current and potential applications of FCS in policy and practice. This review will contribute to the field by providing the first systematic evidence synthesis on FCS, both as a concept and as a practical instrument.

## Project stakeholders

This study is partnered with the RSPB and Natural England. The RSPB and NE partners have been involved in developing the rationale for the review and formulating the review objectives.

### Objectives of the review

This review aims to synthesise and characterise evidence globally to understand how the term FCS is used in the literature and the context of its applications in policy and practice.

Primary question.

- How is FCS defined and how has it been applied in policy and practice?

Secondary questions.

- What barriers or limitations to implementing FCS does the evidence identify?
- Is there geographic clustering or particular scales of studies focussed on FCS?
- Has engagement with FCS changed over time?
- What other conservation assessments or approaches are described as complementary to FCS?

### Question components

The question consists of the following elements following the PCC (Population, Concept, Context) framework:

Population

- Any species or habitat.

Concept

- Favourable Conservation Status definitions and the quantification of FCS through setting Favourable Reference Values (FRVs).

Context

- Conservation policy in the European Nature Directives, the Bonn Convention and the Bern Convention.
- The application of FCS as a scientific conservation assessment framework to inform management decisions within different regions and scales of study.
- Other conservation assessments, such as the IUCN Green Status of Species, may complement FCS as a concept.

## Methods

### Searching for articles

A test list of ten articles and reports known to encompass the different components of the research question was chosen to develop the search strategy. Keywords were recorded from each article and used to develop the search terms. Individual terms were searched to assess the proportional relevance of each term.

The search string was developed in Scopus and began with the fundamental terms “Favourable Conservation Status” and “Favourable Reference Value”, representing the question concepts and their different spellings. The inclusion of keywords representing the question population, such as “species” or “habitat”, and similar concepts to FCS, such as “species recovery”, generated results that were too general to answer the questions. Only articles that used the exact terms FCS/FRVs were deemed relevant to the search, as articles discussing related concepts without using these terms did not meet the eligibility requirements. Therefore, the final search query focused on these fundamental terms in the final search string:

“favourable conservation status” OR “favourable reference values”.

All searches were limited to a date range of 1979-present to reflect the signing of the Bonn Convention, which was later adopted as the Nature Directives in Europe [4, 5].

Backward and forward citation searching of the test list articles was employed using the ‘citationchaser’ application [34]. A manual search of highly related journals was also conducted to uncover additional relevant articles.

Non-English languages will be included in the search by using online translators to translate the search string and the resulting articles found. Further information detailing the search strategy can be found in Additional File 1.

### Bibliographic databases

Peer-reviewed articles will be searched by all fields in the Web of Science Core Collection and Scopus databases. An initial test using these databases returned 741 articles (561 and 180, respectively) (Additional File 1).

The review team has access to two institutional subscriptions from the University of Durham and Newcastle University.

### Web-based search engines

Google Scholar will be used to search for articles by title and by all fields. The title only search should yield the most relevant results to the review. However, to increase the chances of finding more relevant articles, the first 200 articles of the full-text search were also collected. More information on the Google Scholar search, including translated search term results, can be found in Additional File 1 Table 6. This approach should account for publication bias in the literature whilst keeping the search focused and relevant.

### Organisational websites

Relevant webpages from conservation organisations and the 27 Member State’s government and environmental websites will be searched and assessed against the eligibility criteria. The in-website search box will be used to identify webpages from each organisation. Websites will be searched for the term “Favourable conservation status” and sorted by relevance. Google Translate (https://translate.google.co.uk/*)* will be used for websites that are not written in English. The first 25 returns will be taken from the governmental websites and the first 100 returns from all other websites will be assessed. National Biodiversity Strategies and Action Plans (NBSAPs) from the Convention on Biological Diversity (CBD) website (https://www.cbd.int/*)* will also be included.

An initial test search of the government environmental websites yielded 496 web pages, and the conservation organisation websites yielded 240 web pages. Furthermore, 284 NBSAPs were found on the CBD website resulting in 1,020 organisational websites in total. The complete list of websites searched and the number of web pages obtained can be found in Additional File 1.

### Comprehensiveness of the search

The comprehensiveness of the search strategy was assessed using the test list of benchmark articles. If the search terms return all of the articles then the search terms can be deemed comprehensive. When applied to Scopus, Web of Science and Google Scholar, the final search string returned all articles from the test list, indicating a comprehensive search.

ProQuest was considered for both peer-reviewed and grey literature searching, but as this failed to return any of the test list articles (Additional File 1), it will be excluded from the final approach.

The final search across all sources yielded 2,768 articles, of which at least 1,320 were unique.

### Screening process

The screening process will consist of two stages. In the first stage, articles will be screened based on their titles and abstracts. In stage two, retained articles will be screened based on the content within the full text, and a list of articles excluded at the full-text stage with reasons for exclusion provided. Both stages will use the same eligibility criteria as described below.

A screening pilot test was conducted to test the screening strategy and check for required eligibility criteria refinements. A random selection of 25 articles and reports from the collated sources was taken and assessed against the eligibility criteria in a double-anonymised review. After both reviewers completed the screening pilot test exercise, disagreements were resolved through discussion without the need for revising the eligibility criteria.

### Eligibility criteria

Inclusion criteria.

Eligible populations: Species or habitats.

Eligible concept: ‘Favourable Conservation Status’ and ‘Favourable Reference Values’ are referred to in the correct context.

Eligible context: The correct context can be identified if studies link the population and concept to their policy origins, for example, within the Nature Directives. The proper context can also be identified if the concept is used in relation to conservation assessments and management decision frameworks.

Eligibility criteria.

Articles will be assessed against the following eligibility criteria:

Criterion 1: Exclude if FCS/FRV is mentioned incidentally, the words ‘Favourable Conservation Status’ or ‘Favourable Reference Value’ are used in sequence but in an incorrect context.

Criterion 2: Include if the study explicitly focuses on developing methods for setting FRVs.

Criterion 3: Exclude if there is no further discussion or examples of defining FCS, the applications of FCS or the barriers to implementing FCS.

The criteria will be applied in sequence. A hierarchical criteria design ensures that articles are included based on criterion two even if criterion three is met, as we anticipate that methods-focused articles may not meet the other conceptual criteria that criterion three is designed to capture.

### Consistency checking

The review team will comprise a lead reviewer and a second reviewer. A random 20% of articles will be selected for review in stage 1, and all articles will be reviewed in stage 2 (full-text) to check consistency in applying the eligibility criteria. Cohen’s kappa statistic will be used to assess agreement between reviewers. The protocol eligibility criteria will be reviewed if the Kappa statistic is less than 0.6. Full-text coding will also be subject to review by a second reviewer. If articles are too ambiguous to align with the eligibility criteria, these will be marked by the lead reviewer and subject to a second review in addition to the random articles. Any disagreements between reviewers will be resolved through discussion or by including a third reviewer if necessary.

### Reporting screening outcomes

The screening process results will be displayed in a ROSES flowchart diagram [35]. A list of excluded articles with reasons for exclusion will be provided at the second screening stage. A list of the remaining eligible articles will then be presented.

### Data coding strategy

A data coding template has been designed to extract data from the eligible articles (Additional File 2). The coding template was designed to capture the varying components of the primary and secondary research questions such as information on the study country, the scale of study and if and how FCS is defined. If multiple articles refer to the same study, these will be grouped for coding.

### Consistency checking

A coding pilot test was conducted to test the repeatability of the data extraction process when using the coding template. AO and NM independently completed the data extraction process for the ten test list articles. Any inconsistencies were resolved through discussion and, where necessary, the coding categories were refined for clarity.

### Study mapping and presentation

The review will categorise and visualise the body of literature on FCS and include the coded metadata of the eligible articles according to the coding template (Additional File 2). Evidence will be extracted and coded to address the research question components. Visualisations will include a variety of figures and tables to illustrate the distribution of categorical data.

### Narrative synthesis methods

The narrative synthesis will summarise the evidence in a discussion and basic qualitative content analysis, complemented by descriptive statistics and appropriate graphics. Descriptive tables will categorise studies by key characteristics such as feature (species or habitat) focus, geographical location, and methodological approach. Figures may include heat maps, spatial maps, network charts, bar charts and Sankey diagrams to display the connections between coded data to provide evidence relating to the research questions. Figures will also highlight how many papers defined, quantified and discussed the limitations of FCS. Line charts with the number of studies over time will display the temporal patterns in the literature. Tables will include the results of how many studies were found per source.

The potential biases of the methodology will be discussed in the review as it is recognised that the selection process may result in relevant studies being inadvertently excluded.

### Knowledge gap identification strategy

We will use co-occurrence matrices to identify knowledge gaps and clusters by determining which eligible articles provide evidence of FCS being applied, defined, or quantified. To identify evidence on the concept-application gap in the FCS landscape, we will compare the number of studies that propose potential applications of FCS with those providing evidence of FCS being applied in practice. The project stakeholders will be consulted to develop the gap analysis further.

## Electronic supplementary material

Below is the link to the electronic supplementary material.

Supplementary Material 1

Supplementary Material 2

Supplementary Material 3

## Data Availability

No datasets were generated or analysed during the current study.
