# Supplementary material for: What is favourable conservation status?: A systematic map protocol
Source: Environ Evid. 2025 Feb 17;14:3. doi: 10.1186/s13750-025-00356-7 (PMC11834568; doi:10.1186/s13750-025-00356-7)
Supplement: Supplementary file 1 — Supplementary Material 1 [file 13750_2025_356_MOESM1_ESM.pdf]

Search Strategy

Contents

Search Strategy 1
Test-list: Bibliographical information 2
Test-list scope 2
Keywords from test-list 3
Specificity of individual keywords 4
Search string development 5
Non-english search term translations 6
Final search per source 6
SCOPUS 6
Web of Science Core Collection (1900-present) 6
Proquest 7
Google Scholar 7
Organisational Websites 9
Manual search of selected journals 11
Citation search: backward and forward citation searching 11
Total results 11
Performance of the test list 12
Exclusion of proquest database 12

Test-list: Bibliographical information

1) Bijlsma, R. J., Agrillo, E., Attorre, F., Boitani, L., Brunner, A., Evans, P., Foppen, R., Gubbay, S., Janssen, J. A. M., van Kleunen, A., Langhout, W., Noordhuis, R., Pacifici, M., Ramírez, I., Rondinini, C., van Roomen, M., Siepel, H., & Winter, H. V. (2018). Defining and applying the concept of Favourable Reference Values for species and habitats under the EU Birds and Habitats Directives.
2) Bonelli, S., Barbero, F., Zampollo, A., Cerrato, C., Genovesi, P., & La Morgia, V. (2021). Scaling-up targets for a threatened butterfly: A method to define Favourable Reference Values. *Ecological Indicators*, *133*, 108356.
3) Epstein, Y. (2016). Favourable Conservation Status for Species: Examining the Habitats Directive's Key Concept through a Case Study of the Swedish Wolf. *Journal of Environmental Law*, *28*(2), 221–244.
4) Epstein, Y., López-Bao, J. V., & Chapron, G. (2016). A Legal-Ecological Understanding of Favorable Conservation Status for Species in Europe. *Conservation Letters*, *9*(2), 81–88.
5) Green, R., Gilbert, G., Wilson, J., & Jennings, K. (2020). Implications of the prevalence and magnitude of sustained declines for determining a minimum threshold for favourable population size. *PLoS ONE*, *15*(2).
6) JNCC. (2018). Favourable Conservation Status: UK Statutory Nature Conservation Bodies Common Statement. *9*(2), 81–88.
7) Louette, G., Adriaens, D., Paelinckx, D., & Hoffmann, M. (2015). Implementing the Habitats Directive: How science can support decision making. *Journal for Nature Conservation*, *23*, 27–34.
8) Mason, T. H. E., Stephens, P. A., Gilbert, G., Green, R. E., Wilson, J. D., Jennings, K., Allen, J. R. M., Huntley, B., Howard, C., & Willis, S. G. (2021). Using indices of species' potential range to inform conservation status. *Ecological Indicators*, *123*, 107343.
9) Mousley, S., van Vliet, W., & Cork, C. (2023). Defining Favourable Conservation Status in England Natural England approach. Natural England Evidence Information Note EIN062. *Natural England, York*.
10) Trouwborst, A., Boitani, L., & Linnell, J. (2017). Interpreting 'favourable conservation status' for large carnivores in Europe: How many are needed and how many are wanted? *Biodiversity and Conservation*, *26*(1), 37–61.

Test-list scope

Table 1 Details of the geographic scope and type of the test-list articles.

	**Type**	**Country of first author institute**
1	Report	Netherlands
2	Journal article	Italy
3	Journal article	Sweden
4	Journal article	Sweden
5	Journal article	United Kingdom
6	Report	United Kingdom
7	Journal article	Belgium
8	Journal article	United Kingdom
9	Report	United Kingdom
10	Journal article	Netherlands

Keywords from test-list

Table 2 Keywords derived from the test-list articles.

Reference	Keywords
1	**Favourable Conservation Status,** Favourable Reference Population, Habitats Directive,Maxent, *Parnassius apollo*
2	Habitats Directive, **favourable conservation status**, wolf, recovery, Sweden, EU law, FCS
3	Environmental law, **favorable conservation status**, FCS, habitats directive, recovery
4*	BD, Birds Directive, CS, Conservation status, CV, Current value, EC, European Commission, EEA, European Environment Agency, FCS, **Favourable Conservation Status**, FRA, Favourable reference area, FRP, Favourable reference population, FRR, Favourable reference range, FRV, Favourable reference value, HD, Habitats Directive, DV, Directive value, MS, Member State, MVP, Minimum Viable Population, PVA, Population Viability Analysis, SDM, Species Distribution Model
5*	Favourable population size, Minimum threshold, quantifying, unfavourable status, threshold, minimum viable population, sustained declines, **favourable conservation status**
6*	**Favourable conservation status**, favourable reference value, Habitats, species, range, area, population size, structure and function, habitat for the species, future prospects, Favourable, Unfavourable Inadequate, Unfavourable Bad, favourable reference values, FRV
7	**Favourable conservation status,** Management, Measures, Monitoring, Natura 2000, Reference values
8	Baselines, Birds, Conservation status, **Favourable conservation status**, Great Britain, Land use change, Potential ranges, Spatial distributions, Species' distributions, Species distribution models
9	**Favourable conservation status**, defining FCS, FCS methodology, nature recovery
10	**Favourable conservation status,** FCS, Habitats Directive, Large carnivores, Law, Transboundary cooperation

* selected keywords from full-text where list of keywords were not provided in the article

Specificity of individual keywords

Scopus was used to perform a search of individual terms across all fields, limited to 1979-present.

Table 3 Articles returned per search term

**Search term**	**Articles returned**
Species	6,889,941
Habitat	1,165,573
Favourable	813,448
Favorable	813,448
Reference	5,699,496
Value	12,738,745
FCS	35,607
FRV	1,061
Indicators	1,129,303
definition	3,124,750
Range	8,504,092
“natural range”	6,157
Distribution	12,587,735
population	10,233,755
abundance	1,363,485
“population size”	168,971
“habitat quality”	43,011
Structure	22,990,501
function	18,414,143
“structure and function”	1,098,038
{favourable conservation status}	127
{favorable conservation status}	439
**“favourable conservation status”**	532
{favourable reference value}	1
{favorable reference value}	1
{favourable reference values)	41
**“favourable reference value”**	44
“conservation status”	71,874
“reference value”	332,980
Favourable range	303
Favourable reference range	1
Favourable population	43
Favourable reference population	7
“habitat for the species”	327
“species recovery”	1,350
Appl*	14,070,393
Defin*	4,075,771
Interpret*	1,908,137
Implement*	3,676,245
“self sustaining”	9,326
“viable population”	2,439
“minimum viable population”	519
“carrying capacity”	49,557
“MVP”	4,813

Search string development

Table 4 Search string development. Scopus advanced search was used to develop the search string.

String trialled	Search string	Number of results
1	("Favourable conservation status" OR "favourable reference value") AND appl*	444
2	("Favourable conservation status" OR "favourable reference value") AND defin*	177
3	Favourable conservation status OR favourable reference value AND interpret*	192
4	("Favourable conservation status" OR "favourable reference value") OR "species recovery"	7528
5	"Favourable conservation status" OR "favourable reference value" OR "Favourable range" OR "Favourable reference range" OR "Favourable population" OR "Favourable reference population"	939
6	{favourable conservation status} OR {favorable conservation status} OR {favourable reference value*} OR {favorable reference value*}=	529
7*	"favourable conservation status" OR "favourable reference value"	561

* Final search string. Using “” within Scopus allows for different spellings of the words to be identified whereas {} results in only that exact phrase. Therefore the final search string both captures the spelling of ‘favourable’ as ‘favorable’ and cases where ‘value’ is ‘values’.

Non-english search term translations

Table 5 A selection of languages to test the returns per source. Each source was searched by all fields. Terms were translated using Google Translate (https://translate.google.co.uk/).

Language	Term	Web of Science	Google Scholar	Scopus
English	favourable conservation status	183	6,760	565
German	günstiger Erhaltungszustand	0	503	8
Dutch	gunstige staat van instandhouding	0	2,000	2
French	état de conservation favorable	0	1,090	1
Italian	stato di conservazione favorevole	0	197	0
Greek	ευνοϊκή κατάσταση διατήρησης	0	17	0
Romanian	stare de conservare favorabilă	0	76	0
Latvian	labvēlīgs aizsardzības statuss	0	6	0

Terms will be translated for the Google Scholar search only as this yielded the most results for the tested non-English languages in Table 5.

Final search per source

All searches were limited to a date range of 1979-present

SCOPUS

Search string: "favourable conservation status" OR "favourable reference value" by All fields *

*Searching by ALL fields will return documents with the search terms in the article title, source title, language, author, editor, affiliation, abstract, keywords, references, DOI, ISBN, ISSN, CODEN, issue, volume, publication year, sequence bank, sequence bank number, article number, chemical name, CAS registry number, manufacturer, publisher, or conference fields.

Web of Science Core Collection (1900-present)

Search string:

“favourable conservation status" OR "favorable conservation status" OR "favourable reference value*"OR "favorable reference value*" by All Fields

Proquest

"favourable conservation status" OR "favorable conservation status" OR "favourable reference value" OR "favorable reference value"

Results with no filters selected: 602

With peer-reviewed journals selected:113

Grey literature on-page limits selected: 485

- Newspapers
- Dissertations & Theses
- Books
- Historical Newspapers
- Wire Feeds
- Blogs, Podcasts, & Websites
- Other Sources
- Magazines
- Trade Journals

Google Scholar

Google Scholar advanced search was used to search for articles published since 1979 by both title and all fields:

Title

"favourable conservation status" OR "favorable conservation status" OR "favourable reference value" OR "favorable reference value" OR "favourable reference values" OR "favorable reference values" resulted in **3 articles**

All fields

"favourable conservation status" OR "favorable conservation status" OR "favourable reference value" OR "favorable reference value" OR "favourable reference values" OR "favorable reference values" resulted in **7510 articles**

All results from the title search will be used (n=73) plus the first 200 results from the all fields search, resulting in **273 articles.**

Translated terms

The search terms will be translated into each of the official 23 European Member State (non-English) languages (https://european-union.europa.eu/principles-countries-history/languages_en). All results from the title search will be used plus the first 25 results from the all fields search.

The total number of articles found in non-English languages was 37 with the translated search terms in the title and 6,854 with the translated search terms mentioned anywhere in the text (Table 1). This adds 358 articles to the English-language Google Scholar search terms, resulting in 631 articles.

Table 6 Number of results per language returned by Google Scholar, sorted by mentions in title or all fields.

Language	Term	Title results	All field results
Bulgarian	благоприятен природозащитен статус	0	24
Croation	povoljan status očuvanosti	0	4
Czech	příznivý stav ochrany	0	9
Danish	gunstig bevaringsstatus	11	336
Dutch	gunstige staat van instandhouding	8	2,000
Estonian	soodne kaitsestaatus	0	2
Finnish	suotuisa suojelun taso	0	51
French	état de conservation favorable	3	1,090
German	günstiger Erhaltungszustand	4	503
Greek	ευνοϊκή κατάσταση διατήρησης	0	17
Hungarian	kedvező természetvédelmi állapot	0	0
Irish	stádas caomhnaithe fabhrach	0	0
Italian	stato di conservazione favorevole	1	197
Latvian	labvēlīgs aizsardzības statuss	0	6
Lithuanian	palanki apsaugos būklė	0	2
Maltese	stat ta' konservazzjoni favorevoli	0	0
Polish	korzystny stan ochrony	0	13
Portuguese	estado de conservação favorável	0	312
Romanian	stare de conservare favorabilă	0	76
Slovak	priaznivý stav ochrany	0	6
Slovenian	ugodno stanje ohranjenosti	0	26
Spanish	estado de conservación favorable	2	1,070
Swedish	gynnsam bevarandestatus	8	1,110

Organisational Websites

To find relevant organisational websites, we used the governmental and environmental websites of each Member State (MS) country. In addition, we searched European and global conservation websites.

Table 7 Organisational websites per country/region. Total numbers per country are given after each country's rows and totals per grouping are given in bold. *indicates websites where no search bar could be found.

**Region**	**URL**	**Number of results**
Austria	https://www.bmk.gv.at/en.html<br>https://www.umweltbundesamt.at/en/	49<br>33<br>= 82
Bulgaria	https://www.moew.government.bg/en/<br>https://eea.government.bg/en	3<br>0*<br>= 3
Belgium	https://www.belgium.be/en<br>https://www.health.belgium.be/en<br>https://en.vmm.be/	0<br>3<br>4<br>= 7
Croatia	https://vlada.gov.hr/en<br>https://www.haop.hr/ (+'povoljan status očuvanosti')	0<br>1<br>= 1
Cyprus	https://www.moa.gov.cy/moa/environment/environmentnew.nsf/index_en/index_en?OpenDocument<br>https://www.gov.cy/en/ (+' ευνοϊκή κατάσταση διατήρησης')	1<br>0<br>= 1
Czech Republic	https://www.mzp.cz/en<br>https://cenia.gov.cz/czech-environmental-information-agency/#_aktuality	1<br>0*<br>=1<br>= 1
Denmark	https://eng.mim.dk/<br>https://eng.mst.dk/	0*<br>99<br>= 25
Estonia	https://kliimaministeerium.ee/en<br>https://keskkonnaagentuur.ee/en/estonian-environment-agency-news-and-contact/estonian-environment-agency	2<br>1<br>= 3
Finland	https://ym.fi/en/front-page<br>https://valtioneuvosto.fi/en/frontpage	75<br>3<br>= 28
France	https://www.diplomatie.gouv.fr/en/<br>https://www.ecologie.gouv.fr/en (+'état de conservation favorable')	191<br>0<br>= 25
Germany	https://www.bundesregierung.de/breg-en<br>https://www.bmuv.de/en/	0<br>2<br>= 2
Greece	https://www.gov.gr/en/upourgeia/upourgeio-periballontos-kai-energeias	0

	https://necca.gov.gr/en/home/ (+' ευνοϊκή κατάσταση διατήρησης')	0<br><br>= 0
Hungary	https://2015-2019.kormany.hu/en<br>https://portal.cor.europa.eu/divisionpowers/Pages/Hungary-environment.aspx (+kedvező természetvédelmi állapot)	1<br>0<br><br>= 1
Ireland	https://www.epa.ie/<br>https://www.gov.ie/en/organisation/department-of-the-environment-climate-and-communications/	14<br>2<br><br>= 16
Italy	https://www.governo.it/en<br>https://www.isprambiente.gov.it/en	0<br>4<br><br>= 4
Latvia	https://www.mk.gov.lv/en (+'labvēlīgs aizsardzības statuss')	0<br>= 0
Lithuania	https://am.lrv.lt/en/<br>https://gamta.lt/en (+palanki apsaugos būklė)	0<br>0<br>= 0
Luxembourg	https://aev.gouvernement.lu/en.html<br>https://environnement.public.lu/fr.html	0<br>7<br>= 7
Malta	https://environmentcms.gov.mt/en/<br>https://era.org.mt/	3<br>10<br>= 13
Netherlands	https://www.government.nl/<br>https://www.pbl.nl/en	9<br>226<br>= 34
Poland	https://www.gov.pl/web/climate	594,012<br>= 25
Romania	https://www.mmediu.ro/<br>https://www.anpm.ro/	0<br>0<br>=0
Slovakia	https://www.minzp.sk/en/	0*<br>= 0
Slovenia	https://www.gov.si/en/state-authorities/ministries/ministry-of-the-environment-climate-and-energy/<br>https://eurogoos.eu/member/slovenian-environment-agency/ (+' ugodno stanje ohranjenosti)	16<br><br>0<br><br>= 16
Spain	https://www.lamoncloa.gob.es/lang/en/Paginas/index.aspx<br>https://www.miteco.gob.es/es.html	2<br>0<br>=2
Sweden	https://www.government.se/<br>https://www.naturvardsverket.se/en/	0<br>1<br>= 1
United Kingdom	https://www.gov.uk/	17,179 *<br>= 25<br>**MS total = 296**

Europe	European Environment Agency (https://www.eea.europa.eu/<br>European Commission https://commission.europa.eu/index_en	548<br>134,750<br>**= 200**
Global	Internation Panel on Biodiversity and Ecosystem Services (https://www.ipbes.net/)<br>UNEP-WCMC (https://www.unep-wcmc.org/en)<br>BirdLife International https://www.birdlife.org/<br>IUCN (https://www.iucn.org/)<br>WWF https://www.worldwildlife.org/<br>Convention on Biological Diversity (https://www.cbd.int/)	2<br><br>10<br>116<br>99<br>8<br>5<br>**=240**
	→ National Biodiversity Strategy and Action Plans (NBSAPs) post-cop 15 (https://ort.cbd.int/nbsaps)<br>→ NBSAPs Post-COP 10 (https://www.cbd.int/nbsap/about/latest)<br>→ NBSAPs Pre-COP-15	45<br><br>179<br><br>60<br>**= 284**
Total website pages across all organisational websites = 944, capped at 25 results for Member State (MS) country governmental or environmental websites and at 100 for all other websites except for NBSAPs.		**= 1,020**

Manual search of selected journals

**Journal**	**Number of results**
Biological Conservation	84
Biodiversity and Conservation	37
Conservation Biology	27
Journal for Nature Conservation	52
	**Total: 200**

Citation search: backward and forward citation searching

Conducted via https://estech.shinyapps.io/citationchaser/

References from test-list articles (backward citation chasing)	70 records
Citations of test-list articles (forward citation chasing)	122 records
	**Total: 192 records**

Total results

**Database**	**Number of results**
Scopus	561
Web of Science	180
Google Scholar	631
Organisational websites	1,020
Manual search	200
Citation search	192
	**Total: 2,768**

Performance of the test list

Performance of the test list articles. Green: Test list article was returned via that database. Amber: Test list article was not returned via that database.

Reference	Scopus	Web of Science	ProQuest peer-reviewed	ProQuest grey	Google Scholar
(Bonelli et al., 2021)					
(Epstein, 2016)					
(Epstein et al., 2016)					
(Bijlsma et al., 2018)					
(Green et al., 2020)					
(JNCC, 2018)					
(Louette et al., 2015)					
(Mason et al., 2021)					
(Mousley et al., 2023)					
(Trouwborst et al., 2017)					

Exclusion of proquest database

Proquest failed to return any of the test-list articles regardless of the search terms used. We believe that ProQuest does not contain the test-list articles. To test this, we searched the peer-reviewed and grey literature combined (all available articles) specifically for the articles by the first author's name. The test-list articles were not returned using the following searches:

- author(Simona Bonelli) = 3 results, 0 test-list articles
- author(Yaffa Epstein) = 8 results, 0 test-list articles
- author(Rienk Jan Bijlsma) = 0 results
- author(Rhys Green) = 24 results, 0 test-list articles
- author(JNCC) = 0 results
- author(Gerald Louette) = 0 results
- author(Tom Mason) = 110 results, 0 test-list article
- author(Sally Mousley) = 0 results
- author(Arie Trouwborst) = 21 results, 0 test-list articles
