# Supplementary material for: What is favourable conservation status?: A systematic map protocol
Source: Environ Evid. 2025 Feb 17;14:3. doi: 10.1186/s13750-025-00356-7 (PMC11834568; doi:10.1186/s13750-025-00356-7)
Supplement: Supplementary file 3 — Supplementary Material 3 [file 13750_2025_356_MOESM3_ESM.pdf]

| Item | Section / sub-section | Topic | Description | Further explanation | Checklist/Meta-data | Author response | Comments |
|---|---|---|---|---|---|---|---|
| 1 | Title | Title | The title must indicate that it is a systematic map protocol, and must | The title should | Meta-data | What is Favourable Conservation Status?: A Scoping Review Protocol | |
| 2 | Type of review | Type of review | Select one of the following types of review: systematic map, systematic | See CEE Guidance on | Meta-data | systematic map | Systematic map |
| 3 | Authors contacts | Authors contacts | The full names, institutional addresses, and email addresses for all authors | | Checklist | Yes | |
| 4 | Abstract | Structured summary | Abstract must not exceed 350 words and must include two sections 1) | | Checklist | Yes | |
| 5 | Background | Background | Describe the rationale for the review in the context of what is already | A theory of change | Checklist | Yes | |
| 6 | Stakeholder engagement | Stakeholder engagement | The planned/actual role of stakeholders throughout the review process | | Checklist | Yes | |
| 7 | Objective of the review | Objective | Describe the primary question and secondary questions (when | The primary question is | Checklist | Yes | |
| 8 | | Definitions of the question | Break down and summarise question key elements e.g. population, | For other question | Meta-data | Population (Any species or habitat), Concept (Favourable Conservation | |
| | Methods | | | | | | |
| 9 | Searches | Search strategy | | Details regarding | Checklist | Yes | |
| 10 | | Search string | Provide Boolean-style full search string and state the platform for which | | Meta-data | "favourable conservation status" OR "favourable reference value" | |
| 11 | | Languages – bibliographic | List languages to be used in bibliographic database searches. | | Meta-data | English | |
| 12 | | Languages – grey literature | List languages to be used in organizational websites searches and web- | | Meta-data | The EU 24 official languages | |
| 13 | | Bibliographic databases | Provide the number of bibliographic databases to be searched. | | Meta-data | 2 | |
| 14 | | Web – based search engines | Provide the number of web – based search engines to be searched. | | Meta-data | 1 | |
| 15 | | Organisational websites | Provide the number of organisational websites to be searched. | | Meta-data | 61 | |
| 16 | | Estimating the | Describe the process by which the comprehensiveness of the search | | Checklist | Yes | |
| 17 | | Search update | Describe any plans to update the searches during the conduct of the | Optional. A search | Checklist | n/a | |
| 18 | Article screening and study | Screening strategy | Describe the methodology for screening articles/studies for | | Checklist | Yes | |
| 19 | | Consistency checking | Describe clearly the process for checking consistency of decisions | | Checklist | Yes | |
| 20 | | Inclusion criteria | Describe the inclusion criteria used to assess relevance of identified | | Checklist | Yes | |
| 21 | | Reasons for exclusion | State that you will provide a list of articles excluded at full text with | | Checklist | Yes | |
| 22 | Critical appraisal | Critical appraisal strategy | Describe here the method you propose for critical appraisal of study | Optional | Checklist | n/a | |
| 23 | | Critical appraisal used in synthesis | Describe how the information from critical appraisal will be used in | Optional | Checklist | n/a | |
| 24 | | Consistency checking | Describe how repeatability of critical appraisal of study validity will be | Optional | Checklist | n/a | |
| 25 | Data extraction | Meta-data extraction and coding | Describe the method for meta-data extraction and coding for studies | | Checklist | Yes | |
| 26 | Data synthesis and | Narrative synthesis strategy | Describe methods to be used for narratively synthesising the evidence | Vote-counting (tallying | Checklist | Yes | |
| 27 | | Knowledge gap and cluster | Describe the methods to be used to identify and/or prioritise key | | Checklist | Yes | |
| 28 | | Demonstrating procedural | Describe the role of systematic reviewers (who have also authored articles | Reviewers who have | Checklist | Yes | |
| 29 | Declarations | Competing interests | Describe of any financial or non-financial competing interests that the | | Checklist | Yes | None |

**References**


[1] James, K.L., Randall, N.P. and Haddaway, N.R., 2016. A methodology for systematic mapping in environmental sciences. Environmental Evidence, 5(1), p.7.

[2] Bayliss, H.R., Haddaway, N.R., Eales, J., Frampton, G.K. and James, K.L., 2016. Updating and amending systematic reviews and systematic maps in environmental management. Environmental Evidence, 5(1), p.20.

[3] Haddaway, N.R., Kohl, C., da Silva, N.R., Schiemann, J., Spök, A., Stewart, R., Sweet, J.B. and Wilhelm, R., 2017. A framework for stakeholder engagement during systematic reviews and maps in environmental management. *Environmental Evidence* , *6* (1), p.11.

[4] Collaboration for Environmental Evidence. 2018. Guidelines and Standards for Evidence synthesis in Environmental Management. Version 5.0. www.environmentalevidence.org/information-for-authors.

[5] Leeds Institute of Health Sciences. https://medhealth.leeds.ac.uk/info/639/information_specialists/1500/search_concept_tools. Accessed 12/11/2017.
